# Interleukin 13 (IL-13) Signalling as a Potential Target for Cell Therapies in Liver Fibrosis

**DOI:** 10.3390/ijms27062735

**Published:** 2026-03-17

**Authors:** Adam Mazurski, Alicja Bednarz, Piotr Czekaj

**Affiliations:** 1 Department of Cytophysiology, Chair of Histology and Embryology, Faculty of Medical Sciences in Katowice, Medical University of Silesia in Katowice, Medyków 18 Str., 40-752 Katowice, Poland; d201283@365.sum.edu.pl; 2 Students’ Scientific Circle, Department of Cytophysiology, Chair of Histology and Embryology, Faculty of Medical Sciences in Katowice, Medical University of Silesia in Katowice, Medyków 18 Str., 40-752 Katowice, Poland; s91203@365.sum.edu.pl

**Keywords:** liver fibrosis, hepatocytes, non-parenchymal cells, interleukin 13 (IL-13), stem cells, secretome

## Abstract

Liver fibrosis is a regenerative mechanism, but it pathologically intensifies in the course of various diseases, leading to progressive impairment of organ function. This process involves parenchymal cells (hepatocytes) and non-parenchymal cells (Kupffer cells, stellate cells, and endothelial cells). Its classic mechanism is based on the activation of stellate cells, the main effector of fibrosis, by transforming growth factor β (TGF-β), which stimulates excessive collagen production. The role of interleukin 13 (IL-13), which enters the liver parenchyma from resident lymphoid cells, seems to be equally important. By binding to the IL-13Rα receptor on stellate cells, IL-13 initiates their activation and increases the production of type I collagen. This process is supported by the Erk1/2 pathway, which induces the expression of genes promoting extracellular matrix deposition. Due to its role as an initiator of the fibrotic cascade, IL-13 represents a promising therapeutic target for inhibiting progressive scarring. In this context, cell therapies are considered to be of great importance. Mesenchymal and epithelial stem cell secretions contain, among others, exosomes that carry paracrine mediators that can inhibit the profibrotic effects of IL-13 by modulating IL-13 signalling, limiting the development of organ scarring. However, the data on clinical applications of this molecular pathway is scarce, as there are no significant studies focusing on IL-13 influence in liver fibrosis. This review emphasizes the lack of clear clinical data linking the beneficial effects of cell therapy with modulation of the IL-13 pathway, which highlights the need for such studies.

## 1. Introduction

Liver fibrosis is a process that is a component of numerous diseases, both gastroenterological and affecting organs from outside the digestive system. Under physiological conditions, fibrosis is a form of organ regeneration and as such constitutes a normal part of the immune response. By contrast, pathological fibrosis, also known as cirrhosis, which occurs in the course of certain diseases, has a destructive effect. The process of scarring of the liver parenchyma impairs its functioning and leads to progressive organ failure, and indirectly to damage to other organs and systems [1].

Diseases often associated with liver fibrosis include alcoholic steatohepatitis (ASH) and non-alcoholic steatohepatitis (NASH). In these diseases, fibrosis develops secondary to steatohepatitis, an inflammatory form of fatty liver disease, characterised by the accumulation of lipid droplets in the cytoplasm of hepatocytes following cell damage. Fatty liver disease is a reversible process, and with appropriate treatment, including dietary intervention and lifestyle changes, it can be completely reversed [2]. Fibrosis, however, is usually irreversible because the fibrous scar tissue produced by the liver is not easily removed by immune cells [3].

Some patients, especially those with portal hypertension, may develop cirrhotic multiorgan syndrome, which involves the simultaneous failure of multiple organs. The severity of fibrosis, most often assessed using the four-point Meta-analysis of Histological Data in Viral Hepatitis (METAVIR) scale, correlates with patient mortality. These deaths are often caused by oesophageal variceal haemorrhage [4]. In the case of non-alcoholic fatty liver disease (NAFLD), the 10-year mortality rate is twice as high in patients with diagnosed stage 4 fibrosis as in those with stages 0 to 2 [5,6]. A particularly dangerous aspect of liver fibrosis, occurring in any disease that causes it, is the potential for fibrosis to progress to carcinogenesis, which is the classic pathway for the development of hepatocellular carcinoma (HCC) [7].

There are currently no known effective treatment options for reversing the fibrosis process. Although some pharmacological interventions are effective in inhibiting it, particularly the drug combination of pegylated interferon alfa (IFNα) and ribavirin (RBV), used primarily in chronic HCV-induced hepatitis, cases of fibrosis regression are extremely rare [8]. Pharmacological treatment options for liver fibrosis are therefore limited and focus primarily on treating the underlying diseases.

Numerous studies have demonstrated the efficacy of this combination in inhibiting the progression of fibrosis compared to interferon therapy alone [9,10,11,12,13,14], with this therapy having both antiviral and inhibitory effects on stellate cell activity. IFNα has been shown to inhibit transforming growth factor β (TGF-β) protein synthesis and induce stellate cell apoptosis [12]. However, the effectiveness of this therapy is not high (estimated at around 40%), and it is associated with the risk of serious side effects, such as depressive disorders caused by a decrease in serum tryptophan concentration [13].

Preclinical studies (in vitro and in vivo) clearly confirm the key role of interleukin 13 (IL-13) in the pathogenesis of liver fibrosis, which justifiably positions its signalling as a promising target for antifibrotic therapies. The key signalling pathway involves IL-13 production by lymphoid cells, mainly innate lymphoid cells (ILCs), binding of the cytokine to IL-13Rα receptors on the surface of stellate cells, and, as a result, a strong induction of pro-inflammatory and profibrotic genes such as signal transducer and activator of transcription 6 (*STAT6*) and C-X-C motif chemokine ligand 8 (*CXCL8*). This activation results in increased production of TGF-β, which stimulates collagen synthesis.

Clinical trials are being conducted on the use of biological drugs to inhibit liver fibrosis. The primary drugs under consideration are simtuzumab, an antibody targeting lysyl oxidase–like 2 (LOXL2), which is involved in collagen cross-linking with elastin, and pamrevlumab, an antibody against connective tissue growth factor (CTGF). Both drugs are in early testing, and their efficacy is currently assessed as low. Furthermore, biological therapy generates very high financial costs [15,16].

In view of the limitations of current pharmacotherapeutic options, additional methods for inhibiting liver fibrosis are being sought. The growing interest in cell therapies opens up possibilities for their use in the treatment of fibrosis, primarily as an adjunctive therapy.

## 2. The Role of IL-13 in the Mechanism of Liver Fibrosis

Liver fibrosis is a multifaceted process based on at least several parallel signalling pathways within the perisinusoidal space. The classic mechanism of fibrosis involves interactions between hepatocytes, macrophages called Kupffer cells, and myofibroblasts called hepatic stellate cells (HSCs) or Ito cells. The latter, as collagen-producing cells, are the primary effector of fibrosis [17].

**Figure 1:**
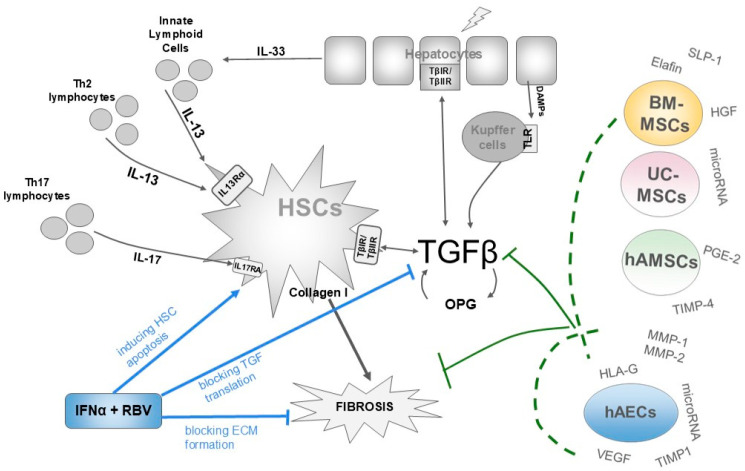
Mechanism of liver fibrosis, including the role of IL-13 and targets for existing drugs and potential cell therapies. Hepatocyte damage triggers the release of interleukin 33 (IL-33), which stimulates lymphoid cells to produce IL-13. After IL-13 binds to IL-13R, STAT6 and AP-1 are activated, inducing *TGF-β* gene expression. Increased TGF-β concentration, in turn, causes the secretion of osteoprotegerin (OPG), which creates a loop that triggers collagen production in stellate cells. Collagen deposition is furtherly enhanced by IL-17 secreted from Th17 lymphocytes via the IL17RA-STAT3 pathway. Classic pharmacological therapy with interferon α and ribavirin increases stellate cell apoptosis and inhibits the formation of TGF-β protein and extracellular matrix. Factors produced by MSCs and hAECs may complement the effects of drugs because they affect similar links in the fibrosis cascade. Their secretome inhibits *TGF-β* and *OPG* gene expression, STAT6 and AP-1 activation, and ECM production. hAECs and MSCs secrete secretomes with partially similar composition but with different proportions of secreted substances. The abbreviations used in the figure are explained in the text.

When hepatocytes and the epithelium of blood vessels or bile ducts are damaged, alarmins, including damage-associated molecular patterns (DAMPs), are released into the liver parenchyma, where they bind to Toll-like receptors (TLRs) on Kupffer cells. In response to the binding of DAMPs to receptors, these cells produce proinflammatory cytokines that affect stellate cells and hepatocytes. The main cytokine produced by Kupffer cells is considered to be TGF-β, which, after binding to receptors and activating SMAD2 and SMAD3 proteins, stimulates stellate cells to secrete extracellular matrix (ECM), rich in type I collagen. This effect is further enhanced by interleukin 17 (IL-17) released from Th17 lymphocytes [17,18]. The second important, and probably key, mechanism responsible for the development of fibrosis is the IL-33/IL-13/TGF-β signalling pathway, in which a relatively recently discovered population of liver lymphocytes, known as ILCs, plays a significant role (Figure 1).

ILCs are constantly present in the liver, but their number is significantly increased in the liver tissue of patients with advanced fibrosis [19]. Dysregulation of specific ILC subtypes is directly linked to the progression of chronic liver diseases (CLD), including metabolic diseases (MASLD/MAFLD) and alcoholic liver disease (ALD). Cytotoxic ILCs (ILC1s) are responsible for immune surveillance and interferon gamma (IFN-γ) production. In chronic inflammatory conditions, their function is often impaired, which promotes progression to HCC. Type 2 ILCs (ILC2s) are considered the main profibrotic population. Clinical studies have shown that their number and degree of activation measured by the expression of markers, e.g., CD69, are significantly increased in the liver tissue of patients with liver fibrosis and cirrhosis. Importantly, ILC2 levels show a strong positive correlation with the degree of fibrosis according to the METAVIR scale. They are a key source of IL-13, which directly stimulates HSCs to produce extracellular matrix. Type 3 ILCs (ILC3s) accumulate in advanced stages of liver cirrhosis. A specific subtype of ILC3s producing IL-13 has been identified in humans, which promotes a pro-inflammatory profile in the organ microenvironment. In metabolic and alcoholic diseases, ILC3s participate in the inflammatory response by secreting IL-17 and interleukin 22 (IL-22), which, depending on the context, can promote regeneration or exacerbate tissue damage [19]. These changes in ILCs numbers open up the possibility of developing new diagnostic methods for liver fibrosis, including morphological analysis of blood for total lymphocyte counts, determination of specific populations using cytometric methods, or quantification of their secretory markers such as IL-17 or IL-22.

**Figure 2:**
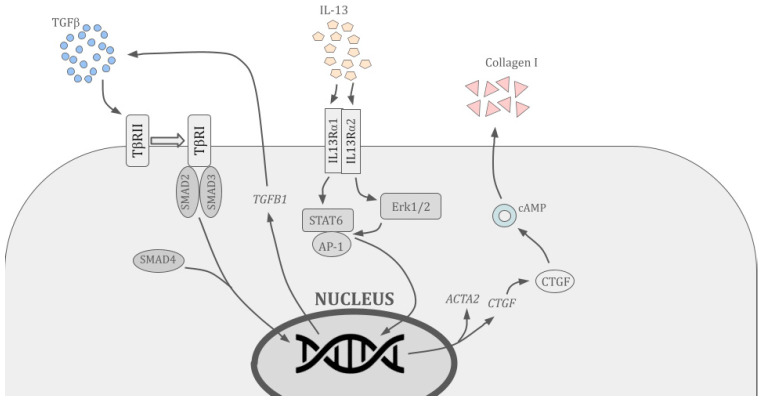
Molecular basis of the interaction between IL-13/TGF-β and collagen production by hepatic stellate cells. Binding of TGF-β via the TβRI receptor activates the TβRII receptor, which phosphorylates SMAD2 and SMAD3 proteins. These form a complex with SMAD4, which translocates to the nucleus and induces expression of the *ACTA2* and *CTGF* genes. CTGF induces the production and secretion of collagen I into the environment via cAMP. Binding of IL-13 via IL-13RaI activates the STAT6-AP-1 complex, which induces expression of the TGFB1 gene in the nucleus. The resulting TGF-β participates in a positive feedback loop.

In response to damage, hepatocytes secrete IL-33 into the environment, which binds to the suppression of tumorigenicity 2 receptors (ST2) on the surface of the ILCs, stimulating them to produce IL-13. The latter is a ligand for the membrane IL-13 receptor alpha (IL-13Rα), which in turn is a subunit of the common receptor for IL-13 and interleukin 4 (IL-4), present on stellate cells, which in this configuration are both effectors and mediators of fibrosis. IL-13, via Erk1/2 proteins, induces *IL-13* gene expression and triggers the production of type I collagen, which is deposited in the liver parenchyma, where it forms fibrous tissue [20,21,22]. Moreover, binding of IL-13 by IL-13Rα has been shown to activate STAT6 and activator protein 1 (AP-1), which in turn induce *TGF-β* gene expression (Figure 2). Binding of IL-13 also induces the production of osteoprotegerin (OPG), the concentration of which in liver tissue correlates with the severity of fibrosis and which increases the production of TGF-β and extracellular matrix in HSCs [20,23].

The existence of IL-13/TGF-β pathway has been demonstrated in numerous preclinical studies focusing on the pathogenesis of liver fibrosis. The concentration of IL-13 produced by ILCs positively correlates with the Model for End-Stage Liver Disease (MELD) score, which reflects the degree of liver damage. IL-13 also induces the expression of pro-inflammatory genes, including *CXCL8* in stellate cells, which leads to increased monocyte migration and promotes the perpetuation of inflammation [24]. The importance of IL-13 in the development of nonalcoholic fatty liver disease is also supported by clinical evidence [25], which has shown a positive correlation between serum IL-13 levels and the degree of fatty liver disease. This also indicates the role of chronic IL-13-mediated inflammation in the progression of fatty liver disease and cirrhosis. Moreover, IL-13 is associated with high levels of inflammatory CD45^+^ cells and α-SMA^+^ myofibroblasts in biliary atresia (BA), in conjunction with effector protein, periostin [25].

It has also been demonstrated that elevated serum IL-13 levels not only promote the development of cirrhosis, but are associated with an alternative pathway of HCC de novo development, in which carcinogenesis occurs without significant underlying fibrosis [26]. Elevated levels of IL-13 are also positively correlated with the likelihood of developing HCC in nonalcoholic steatohepatitis (NASH) [27] and hepatitis C [28]. This may be related to the proven influence of IL-13 on the development of other cancers, specifically myeloproliferative syndromes, including myelofibrosis. It has been shown that elevated IL-13 levels induce TGF-β and collagen production in megakaryocytes and bone marrow–derived mesenchymal stem (BM-MSCs), and that knockout of the gene for the common IL-13/IL-4 receptor reduces the degree of bone marrow fibrosis [29].

Although both IL-13 and TGF-β are important and reliable markers of liver fibrosis, currently, no guidelines recommend their routine measurement in blood serum for diagnostic purposes and disease monitoring. This is consistent with the current state of knowledge in laboratory diagnostic testing, where stable and inexpensive markers such as C-reactive protein (CRP) and D-dimer are routinely used to measure inflammation, rather than specific cytokines such as interleukin 6 (IL-6) or tumor necrosis factor alpha (TNFα). Highlighting the role of IL-13 in clinical settings provides a strong rationale for the development of potential therapies that could target the suppression of IL-13 signalling in the liver microenvironment. It is possible that with the development of laboratory techniques, cytokine measurement will become cheaper and more widely used, allowing IL-13 or TGF-β to be used as useful markers of liver disease.

## 3. Stem Cell Therapy for Liver Fibrosis Targeting IL-13 Signalling

Stem cells have long been recognized as an important component in the treatment of cancer, traumatic injuries, and autoimmune diseases. Their common feature is a low degree of differentiation, pluripotency, or multipotency, which means the ability to differentiate into various types of mature cells. While haematopoietic cell therapies have a well-established role in the treatment of haematological diseases and cancers, the clinical application of non-haematopoietic cells is a relatively new and not fully explored concept [14,30,31,32,33,34]. Among them, significant attention is paid to multipotent stromal cells, previously collectively called mesenchymal stem cells (MSCs), and human amniotic epithelial cells (hAECs) [35]. Depending on their occurrence, MSCs are divided into subtypes, such as stem cells from bone marrow (BM-MSCs), adipose tissue (ADSCs), umbilical cord (hUC-MSCs/hUC-SCs), and amnion (hAMSCs) [36]. These are the most common locations from which MSCs are obtained, due to the availability of source tissues, the lack of strict ethical restrictions, and the ease and high efficiency of isolation [37]. All MSCs share low immunogenicity, expressed by near-zero expression of major histocompatibility complex class II (MHCII) molecules, which significantly increases their safety as a therapeutic agent [38]. Compared to mesenchymal cells, epithelial, ectodermal hAECs are characterised by very low telomerase activity. This makes them potentially more long-lived, but limits their proliferation rate [39]. In in vitro cultures, hAECs easily undergo epithelial–mesenchymal transition (EMT), as a result of which they acquire a phenotype and cytochemical profile typical of MSCs [40]. In the amniotic membrane hAECs constitute a significantly smaller population as compared to mesenchymal hAMSCs. hAECs are characterized by the expression of cell markers such as cytokeratins, E-cadherins, CD9, and human leukocyte antigen G (HLA-G), while hAMSCs are CD73-, CD90-, and CD105-positive, with both cell types exhibiting partially overlapping secretory profiles [41].

### 3.1. Characterization of the Stem Cell Secretome

A significant advantage of stem cells is their ability to release a secretome containing cytokines, growth factors, and nucleic acid fragments, which are known to modulate cellular pathways involved in organ fibrosis and inhibit inflammatory processes in the local cellular microenvironment. There are significant differences between the secretome composition of MSCs and hAECs, even if they originate from the same organ. A new study by Zhang et al. (2025) provides key evidence of significant differences in the secretions of hAECs and hUC-MSCs, which may have a direct impact on their potential efficacy in inhibiting liver fibrosis [42]. The hUC-MSC cell secretome is rich in cytokines that inhibit the activity of lymphocytes and macrophages and reduce the level of proinflammatory mediators (such as TNF-α). Factors found in MSC secretomes include well-known anti-inflammatory and immunomodulatory compounds such as interleukin 10 (IL-10), prostaglandin E2 (PGE2), hepatocyte growth factor (HGF), elafin, and secretory leukocyte protease inhibitor (SLPI), the latter two also exhibiting antibacterial activity [16,43,44]. The MSC secretome also contains tissue inhibitors of metalloproteinases (TIMPs), mainly TIMP4, which reduce ECM production, as well as microRNA and tRNA fragments released by exosomes, which can regulate the expression of genes responsible for fibrosis in effector cells [45,46].

On the other hand, the hAEC secretome is characterized by higher concentrations of proangiogenic factors, e.g., vascular endothelial growth factor (VEGF) and proteins that support structural tissue remodelling. These differences may determine the timing of therapy for liver fibrosis. Potential personalized therapy may be most effective at a specific stage of the disease in treating liver fibrosis: hUC-MSCs in the active inflammatory phase (blocking profibrotic signals such as IL-13) and hAECs in the regenerative phase (rebuilding organ structure). Another possible approach is to use a combination of secretomes from both populations to achieve a synergistic effect—simultaneously suppressing inflammation and stimulating tissue repair. In summary, the variability of the secretome composition derived from stem cells with different genotypes and phenotypes should be taken into account, which is why there is no standardized "fingerprint" for the secretome composition that could affect the reproducibility of preclinical and clinical test results.

As of December 2025, a search of PubMed, ResearchGate, and ClinicalTrials.gov identified a total of four preclinical studies and four clinical trials supporting the therapeutic effects of MSCs in liver fibrosis and their impact on factors associated with IL-13 signalling. These studies highlight the therapeutic potential of MSC-released secretions and extracellular vesicles as modulators of molecular mechanisms adjacent to the IL-13 pathway. To date, one study has been published on hAECs in the context of liver fibrosis.

### 3.2. Preclinical Studies of Cell Therapy for Liver Fibrosis with Potential Impact on IL-13 Signalling

Preclinical studies have confirmed that stem cells derived from sources such as bone marrow or human umbilical cord exert potent antifibrotic effects in liver fibrosis models, partly by modulating cytokine pathways related to IL-13. The most important links between IL-13 activity and the function of these cells are summarised in Table 1. Included studies combine the use of in vivo and in vitro models [46,47,48,49].

hUC-MSCs demonstrate the ability to inhibit HSCs activation by downregulating TGF-β1 expression [46]. At the same time, they suppress key profibrogenic signalling pathways, such as TGF-β/Smad/RhoA/ERK, supporting anti-inflammatory and regenerative processes [47]. Given that IL-13 acts synergistically with TGF-β, in part through shared signalling pathways, to activate stellate cells and induce collagen secretion, this suggests the possibility of indirectly suppressing IL-13 effects through paracrine cell activity.

BM-MSCs alleviate liver fibrosis by reprogramming macrophages to an anti-inflammatory phenotype. As a result, they increase the secretion of IL-4 and IL-10, which leads to reduced expression of profibrogenic cytokines (TGF-β, TNF-α) [49]. IL-13 belongs to the same Th2 cytokine family as IL-4 and exhibits similar molecular effects. It can therefore be assumed that by influencing macrophages, cell therapies could also modulate the activity of the IL-13 axis in the hepatic microenvironment. In a non-hepatic allergic inflammation model, BM-MSCs have the ability to reduce STAT6 expression at the mRNA and protein levels [50]. Given that STAT6 is a key protein phosphorylated upon IL-13Rα1 activation, a potential therapy could act as an inhibitor of the IL-13/STAT6 pathway, limiting the activity of this cytokine. This mechanism makes MSC secretome a promising tool for suppressing pathological IL-13 signalling not only in allergic diseases but also in other conditions where its pathway contributes to fibrosis or chronic inflammation, particularly fibrotic liver diseases.

The collected data indicate that MSCs and their secretome affect key components of inflammatory and fibrogenic cascades converging with IL-13 signalling. Consequently, engineering MSCs (for example, to secrete antagonists of this interleukin) or combination therapies represent a promising direction for further research.

Some publications indicate that hAECs may have a protective effect against fibrosis in various organs. These cells have been shown to reduce inflammation, oxidative stress, and the proliferation of liver progenitor cells (LPCs) [51]. The use of both the cells and their exosomes also leads to a reduction in myocardial fibrosis [52]. In addition, hAECs enhance angiogenesis by increasing VEGF-A expression and reduce collagen deposition by increasing matrix metalloproteinase-8 (MMP-8) expression, including in uterine scar tissue [53].

### 3.3. Clinical Studies of Cell Therapy for Liver Fibrosis with Potential Impact on IL-13 Signalling

There are currently no clinical trials aimed at monitoring the IL-13 axis. The analysed scientific publications focus on assessing the safety and efficacy of hUC-MSCs and hAECs in the treatment of fibrosis. However, these therapies have been shown to affect the concentrations of selected cytokines, including interleukin 8 (IL-8), whose concentration is correlated with IL-13 in cancer. This suggests the ability to modulate the cytokine profile, including IL-13 [54]. Table 2 summarises the studies and observed effects of cell therapies in patients with diseases involving liver fibrosis. These interventions improve liver function parameters and increase both long-term and 6-month survival while maintaining a favourable safety profile [54,55,56,57,58].

The confirmed efficacy of MSCs and hAECs in improving the hepatic microenvironment and their effect on cytokine concentrations indicate their potential as a therapeutic platform that can be specifically targeted at IL-13. The main conclusions were drawn from biochemical and clinical parameters, as well as survival analysis; none of the studies included histopathological assessment of the liver. Administration of hAECs is safe and well tolerated, but their potential anti-inflammatory and antifibrotic effects require confirmation in larger studies [58]. Long-term clinical trials involving large numbers of patients are necessary to directly assess IL-13 expression levels and the activity of its signalling pathway.

## 4. Conclusions

Liver fibrosis is a serious and widespread clinical problem. Key issues that need to be addressed include the limited efficacy of standard pharmacotherapy, the serious side effects of conventional medications, and the limited evidence for the usefulness of new biological drugs. Our goal was to highlight the IL-13 signalling pathway as a potential target for cell-based therapies. There is an urgent need for preclinical and clinical studies that focus on the influence of the stem cell secretome on the concentration of IL-13 and other signalling elements involved in this pathway.

Classic pharmacological therapy with interferon α and ribavirin increases stellate cell apoptosis and inhibits the synthesis of TGF-β protein and extracellular matrix. Currently, there are no studies demonstrating the use of anti-IL13 antibodies in the treatment of liver diseases. Although such drugs include dupilumab, lebrikizumab, and tralokinumab, which are used to treat asthma and atopic dermatitis [59,60], there is no information on their use in the treatment of liver fibrosis or other hepatological diseases [21]. On the other hand, factors produced by MSCs and hAECs can inhibit *TGF-β* and *OPG* gene expression, AP-1 activation, and collagen production, which reduces the severity of fibrosis. Numerous lines of evidence suggest that the secretome of hUC-MSCs and BM-MSCs effectively inhibits the profibrotic effects of IL-13. Bioactive factors produced by these cells significantly reduce STAT6 expression and inhibit the expression of TGF-β and type I collagen genes, thereby contributing to macrophage polarisation towards anti-inflammatory phenotypes. All these effects combine to block the IL-13 pathway. In addition to being a promising source of antifibrotic factors, epithelial hAECs may represent a valuable component of regenerative therapies for fibrosis thanks to their elevated HLA-G expression and production of angiogenic factors.

Although stem cells exhibit immunomodulatory, anti-inflammatory, and regenerative effects, they do not eliminate the underlying cause of the disease or fully reverse advanced scarring. Therefore, their most rational use is as a complementary therapy to primary treatments targeting the cause and key pathways of fibrogenesis. Currently, there is no clinical evidence linking the efficacy of non-haematopoietic stem cell therapy in liver diseases with direct inhibition of IL-13 signalling. Given the documented profibrogenic effects of this interleukin and the expanding clinical evidence supporting the safety and efficacy of stem cells, the development of new therapeutic strategies combining stem cells with targeted IL-13 modulation is increasingly justified.

## Figures and Tables

**Table 1:** Mechanisms by which MSCs and their secretome may modulate different components of the IL-13 axis in liver fibrosis. Green arrow (up) means increase and the red arrow (down) means decrease.

Cell Type/Secretome	Mechanism of Action of MSCs and Their Secretome	Major Observed Biological Effects	Potential Effect on IL-13 Signalling	Ref.
hUC-MSCs	Reduction in TGF-β1 and COL1A1 levels and restoration of MMP-1/TIMP-1 balance	**↓** activation of HSCs **↓** proliferation of activated lymphocytes **↓** IL-6 and IL-8 Improvement of biochemical indicators (ALP/ALB)	Reduction of TGF-β-the main effector of IL-13-induced fibrogenesis	[46]
hUC-MSCs (Wharton’s Jelly)	Inhibition of the TGF-β/Smad/RhoA/ERK pathway	**↓** expression of plasma fibronectin (pFN) **↓** activation of profibrogenic cascades **↑** HGF, PCNA	Reduced response of HSCs to TGF-β	[47]
	Delivery of miR-148a-5p via extracellular vesicles to HSCs	**↓** expression of SLIT3 **↓** activation of HSCs **↓** COL1A1 and α-SMA	Weakening the action of IL-13 effects by inhibiting the expression of ECM-related genes	[48]
BM-MSCs	Conversion of Ly6Chi macrophages into Ly6Clo macrophages	**↓** TGF-β, PDGF, TNF-α regression of fibrous scarring **↓** activation of HSCs **↑** degradation of ECM	Influence on IL-13 signalling by modulating the common Th2 cytokine environment (IL-4, IL-10)	[49]

**Table 2:** Application of hUC-MSCs, hUCB-MSCs, and hAECs in the treatment of diseases associated with liver fibrosis–clinical trials. hUCB-MSCs, human umbilical cord blood-derived mesenchymal stem cells. Green arrow (up) means increase and the red arrow (down) means decrease.

Cell Type	Disease	Route of Administration	Duration of Cell Administration	Clinical and Laboratory Effects	Survival	Adverse Effects	Ref.
hUC-MSCs (Wharton’s Jelly)	HBV-related decompensated cirrhosis	Intravenous	15 days	**↑** albumin **↓** IL-8 Improvement of PTTA and AT-III	Improved 6-month survival	Mostly mild adverse effects	[54]
			8 weeks	**↑** albumin **↑** prothrombin	Improved long-term survival	No serious adverse effects	[55]
	HBV-related liver failure and cirrhosis		4–8 weeks (group-dependent)	Liver failure: **↓** ALT, AST, TBIL, MELD **↑** PTA Cirrhosis: **↓** ALT, AST, TBIL, no changes in MELD, PTA	Not analysed	No serious adverse effects	[56]
hUCB-MSCs	Decompressed cirrhosis	Not specified	Not specified	No evidence of increased HCC risk	Improved long-term (3- and 5-year) survival	Not analysed	[57]
hAECs	Decompressed cirrhosis	Intravenous	Up to 28 days (group-dependent)	**↓** FIB-4 **↓** AST ALT, MELD, HVPG without a clear improvement trend	Not analysed	No serious adverse effects	[58]

## Data Availability

No new data were created or analyzed in this study. Data sharing is not applicable to this article.
